# Toward Integration of mHealth in Primary Care in the Netherlands: A Qualitative Analysis of Stakeholder Perspectives

**DOI:** 10.3389/fpubh.2019.00407

**Published:** 2020-01-15

**Authors:** Esmee L. S. Bally, Tomris Cesuroglu

**Affiliations:** ^1^Faculty of Science, Athena Institute, Vrije Universiteit Amsterdam, Amsterdam, Netherlands; ^2^Department of Public Health, Erasmus University Medical Centre, Rotterdam, Netherlands

**Keywords:** mHealth, chronic disease management, personalized health, health system integration, stakeholder analysis, participatory research, transdisciplinary research

## Abstract

**Background:** There is a growing need to structurally change the way chronic illness care is organized as health systems struggle to meet the demand for chronic care. mHealth technologies can alter traditional approaches to health care provision by stimulating self-management of chronically ill patients. The aim of this study was to understand the complex environment related to the introduction of mHealth solutions into primary care for chronic disease management while considering health system functioning and stakeholder views.

**Methods:** A transdisciplinary approach was used informed by the Interactive Learning and Action (ILA) methodology. Exploratory interviews (*n* = 5) were held with representatives of stakeholder groups to identify and position key stakeholders. Subsequently, professionals and chronically ill patients were consulted separately to elaborate on the barriers and facilitators in integration, using semi-structured interviews (*n* = 17) and a focus group (*n* = 6). Follow-up interviews (*n* = 5) were conducted to discuss initial findings of the stakeholder analysis.

**Results:** Most stakeholders, in particular primary care practitioners and patients, seem to have a supporting or mixed attitude toward integration of mHealth. On the other hand, several powerful stakeholders, including primary care information system developers and medical specialists are likely to show resistance or a lack of initiative toward mHealth integration. Main barriers to mHealth integration were a lack of interoperability with existing information systems; difficulties in financing mHealth implementation; and limited readiness in general practices to change. Potential enablers of integration included co-design of mHealth solutions and incentives for pioneers.

**Conclusion:** Stakeholders acknowledge the benefits of integrating mHealth in primary care. However, important barriers perceived by end-users prevent them to fully adopt and use mHealth. This study shows that the complexity of introducing mHealth into primary care calls for strategies encouraging collaboration between multiple stakeholders to enhance successful implementation.

## Introduction

Management of chronic diseases has become a major priority for healthcare systems around the world (1, 2). The rising prevalence of chronic diseases combined with severe shortages of medical staff pressures the ability of systems to meet the demand for chronic care services (2, 3). Given these circumstances, many Western countries implemented policies to make the shift from an acute model of care toward an integrated and proactive approach to chronic disease management (4). One such a comprehensive approach is the chronic care model (CCM).

Since 2008, the CCM has a guiding role in chronic disease management in the Netherlands (5, 6). This evidence-based framework is recognized for its ability to improve care processes and clinical outcomes (1, 7). The model is structured around a number of elements that encourage productive interactions between integrated healthcare teams and patients (1). An important element to achieve this constructive patient-professional relationship is the support for patient self-management. Self-management enables an individual to cope with his or her disease. This includes the ability to manage symptoms, make lifestyle changes and adhere to treatment regimen (8).

The effect of self-management on the quality of chronic care has been studied extensively in recent years. Research has shown that patients who actively engage in their own care experience improved self-efficacy and better quality of life (9–11). Mobile health (mHealth) can be effective to stimulate self-management (12). In contrast to eHealth (electronic Health), which refers to the general use of information and communication technologies for health, mHealth encompasses a spectrum of mobile technologies aimed at facilitating the collection and communication of health data to improve healthcare service delivery processes (12, 13). This includes technologies such as mobile phones, personal digital assistants (PDA), smartphones, video-game consoles, and handheld computers (12, 13). These devices can be used to send text messages, share photos and video, enable conversations, access the World Wide Web, and support software application (12–14). Typically, mHealth interventions use tools for self-monitoring (e.g., graphs), reminders to perform self-care behaviors (e.g., to take medication), motivational messages, and educational material (14). It increases patient ownership by allowing users to have insight in their health data. Additionally, mHealth fosters collaboration by facilitating communication between patients and healthcare providers (13, 15).

Despite its potential, difficulties in implementation prevent mHealth solutions to be fully embedded in real-world settings (15). A recent study on the readiness of patients with congenital heart disease to adopt mHealth in their care shows the majority to be willing to use mHealth (16). However, only a small portion used mHealth in their care.

To date, there have been various pilots on mHealth in chronic care, but scale-up has not taken place (16–18). In order to successfully introduce mHealth in chronic care, critical stakeholders need to be engaged (15, 16). Particularly, involving end-users, such as patients and practice nurses engaged in chronic care, is crucial to achieve wide-spread integration. Furthermore, the overall health system needs to be well-understood with a systematic approach in order to understand its dynamics that lead to barriers and facilitators for mHealth integration (19, 20).

This study aims to understand the complex environment related to the introduction of mHealth solutions into primary care in the Netherlands in order to explore strategies for integration while considering health system functioning and stakeholder views. In seeking strategies to support integration, barriers, and facilitators in the integration of mHealth were examined systematically (20). Exploring stakeholder positions and views, including end-user perspectives will contribute to better-informed and more effective integration strategies.

## Materials and Methods

### Research Design

The exploratory research was initiated by a medical technology company and conducted in partnership with a university institute specialized in participatory methodologies and health systems. The medical technology company contributed to the research by representing the mHealth developers' perspective. A transdisciplinary approach, informed by the Interactive Learning and Action (ILA) methodology (21), was selected to unravel the barriers and facilitators that influence mHealth integration and to explore the stakeholders engaged in this process. ILA is a well-established methodology characterized by a multi-stakeholder dialogue process aiming to enhance knowledge integration (22). This particular strength of ILA aligned with the focus of this research to engage multiple stakeholders, including health policymakers, health insurers, primary care professionals, and chronically ill patients.

**Figure 1:**
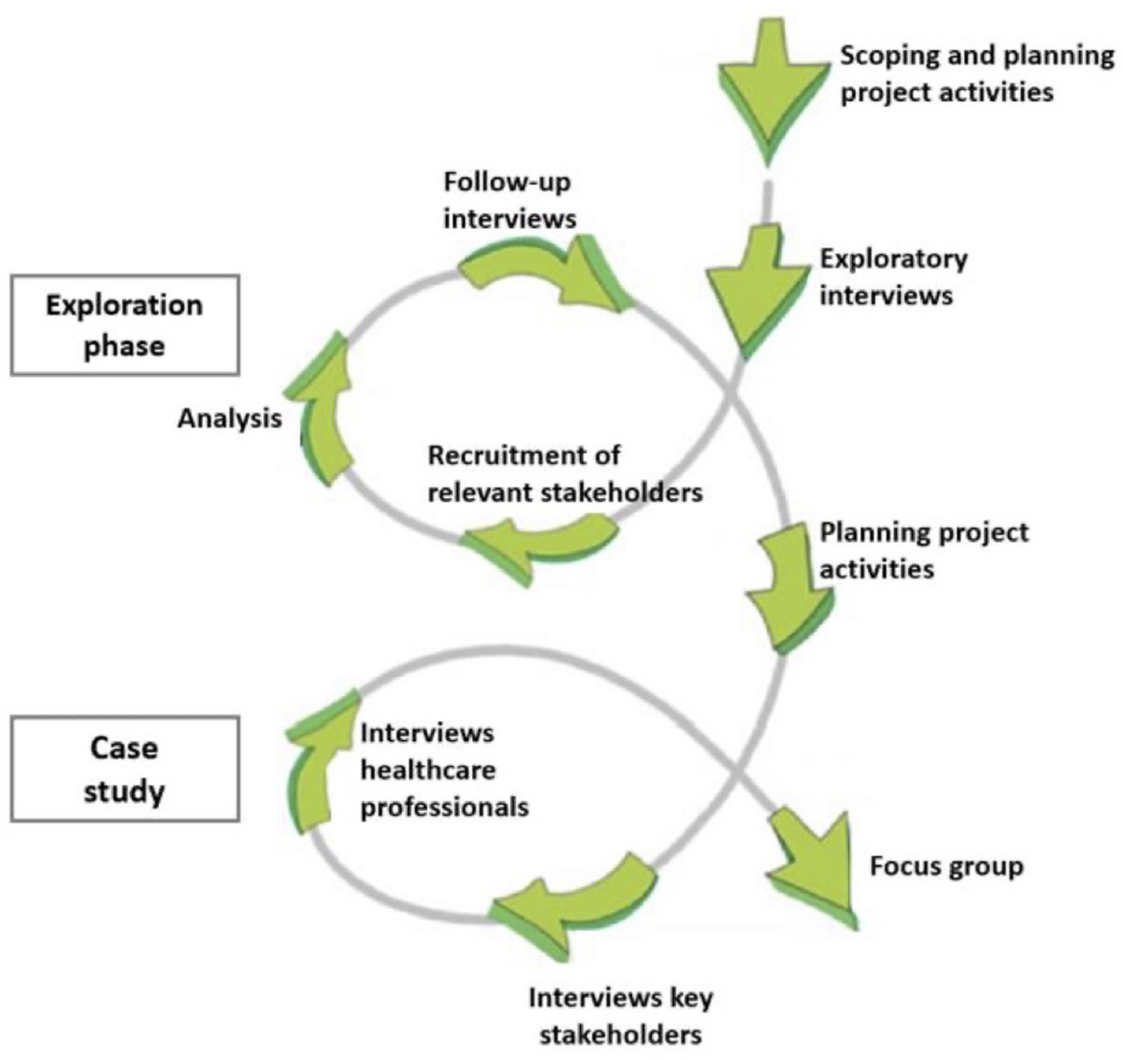
Learning cycles in the research process.

The process of ILA is structured according to five phases: exploration, consultation, integration, prioritization, and implementation (22). Activities are guided by the learning-action spiral of recurring activities of planning, action, observation, reflection, re-planning, etc. (Figure 1). Ideally, studies using the ILA methodology complete several learning cycles in which participants reflect on research outcomes and provide suggestions for adjustments in project activities (23). However, in the context of this exploratory study the focus has been on exploration and consultation.

In this study, key characteristics of the first three phases of ILA were applied to the Dutch primary care context and provided a sound basis to understand health system functioning and considering stakeholder views related to future mHealth integration. The last two phases of the ILA approach were not within the scope of this research and therefore not applied. Interviews and a focus group were used to support participation, dialogue and reflection among the stakeholders. Interviews were considered a suitable method to stimulate reflection and to gain in-depth knowledge of several stakeholders. A focus group was held to encourage dialogue and to include experiential knowledge (24, 25). Table 1 provides an overview of activities conducted in this study.

**Table 1:** Activities structured per project phase.

**Activities**
**PHASE 1: PREPARATION AND EXPLORATION (MAY–AUGUST 2017)**
• Scoping meetings with partner organization to determine research objectives
• Study design was reviewed and approved by the Internal Committee Biomedical Experiments of Philips Research, Netherlands
• Exploratory interviews (*n* = 5) with representatives of the Ministry of Health, the national GP association, a health insurance company and patient organizations to identify stakeholders engaged in future integration of mHealth
• Recruitment of participants for interviews and the focus group
**PHASE 2: DATA COLLECTION (NOVEMBER 2017–APRIL 2018)**
• In-depth interviews (*n* = 6) with professional stakeholders from government and insurance companies to explore views on the barriers and facilitators in the integration of mHealth in primary care
• Primary care professionals (e.g., GPs, practice nurses, and managers) were consulted (*n* = 12) regarding their work situation and shared their perspectives on the barriers and facilitators in the adoption of mHealth solutions
• The needs and perspectives of chronically ill patients were explored using a focus group (*n* = 6)
**PHASE 3: ANALYSIS AND INTEGRATION (JANUARY–SEPTEMBER 2018)**
• Perspectives of different stakeholders were analyzed and compared • Perspectives of primary care professionals obtained through interviews were presented and discussed in the patient focus group • Results of the stakeholder analysis were discussed with participants at exploratory interviews to validate findings (*n* = 5)

The first phase comprised exploratory interviews to identify stakeholders involved in the integration of mHealth in primary care (Table 1). Findings of the exploratory interviews informed the recruitment of key stakeholders. In the third phase, a learning cycle was established by a follow-up of post-interviews with participants at exploratory interviews to present and discuss the findings of the stakeholder analysis (Figure 1, exploration phase).

The second phase included in-depth interviews with key stakeholders to examine the barriers and facilitators in the integration of mHealth in primary care (Table 1). Additionally, semi-structured interviews were held with primary care professionals. A second learning cycle started by sharing the perspectives of this particular end-user group in the focus group with chronically ill patients (Figure 1, case study). As a heterogeneous dialogue meeting of chronically ill patients and primary care professionals was not possible, integration of perspectives, and thus mutual learning was encouraged through this method.

### Analytical Approach

To assess the estimated power and interest of stakeholders, three levels were used based on Covey's circle of concern/circle of influence (26):

1. control (i.e., the stakeholder has the ability to control the adoption of innovations, can prevent further integration or help making it happen);
2. influence (i.e., the stakeholder has the ability to influence developments with regard to the adoption of innovations; less control but important to realize or prevent integration);
3. interest/concern (i.e., the stakeholder is interested in the adoption of innovations or concerned but has no significant ability to impact integration) (26, 27).

**Figure 2:**
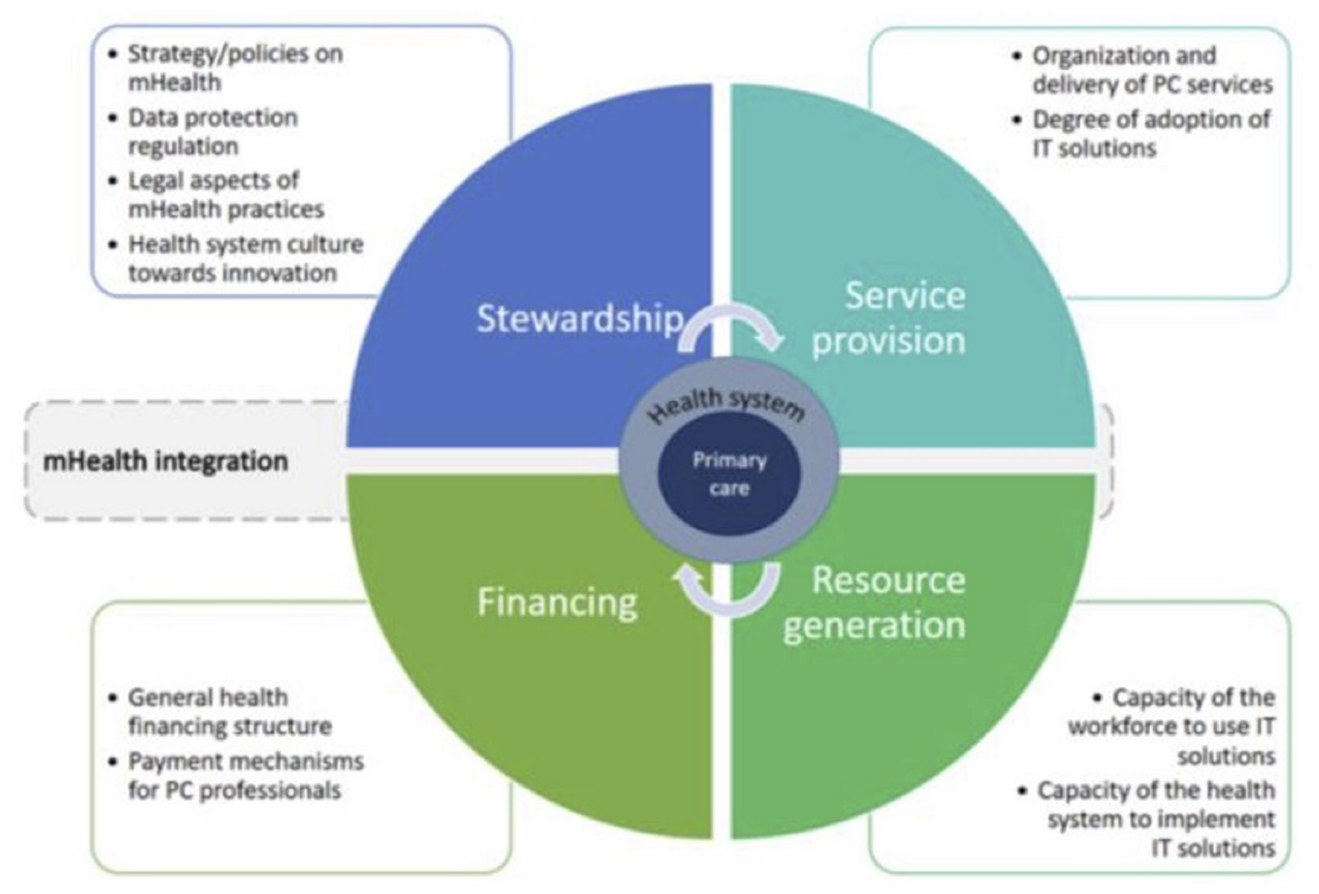
Analytical approach to assessment of the health system for the integration of mHealth (18, 19).

For assessment of the health system, a modified version of Murray and Frenk's model for assessing the performance of health systems was used [Figure 2; (19, 20)]. Functions of a health system, namely stewardship, financing, service provision and resource generation as defined by Murray and Frenk, provided the broad framework for approaching the health system in a comprehensive way. Furthermore, potential areas to explore within these functions were identified at the design stage of the study with the help of an earlier research which explored integration of an innovation into primary care services (20).

### Setting

Data collection took place between May 2017 and September 2018 in the Netherlands. Face-to-face or, when preferred, telephone interviews with key stakeholders were held within the national scope of the study. Nine general practices agreed to participate in the research. Due to a lack of time of GPs, practice nurses and managers, semi-structured telephone interviews proved a feasible method to include this stakeholder group. General practices were located both in urban and rural areas and consisted of solo and group practicing GPs. The patient focus group was held in a rural area, however, through Skype one chronically ill patient from an urban area was able to join the focus group.

### Participants

Participants of the interviews were recruited based on purposive sampling to achieve maximum variation. Convenience sampling was used to recruit patients for the focus group.

#### Representatives of Stakeholder Groups

Exploratory face-to-face interviews (*n* = 5) were held with a representative of the national patient organization, the heart foundation, the national GP association, the Ministry of Health and a health insurance company. The chosen participants were of high representative value as each of them reflected the view of the community they represent. Following inclusion criteria were applied: (1) representatives worked at organizations or institutions representing key stakeholder groups, including chronically ill patients, GPs, health policymakers and health insurers; (2) representatives held a management or policymaking position in mHealth, health innovation or an equivalent; and (3) representatives had sufficient knowledge of the Dutch health system. Representatives with <6 months experience in the mHealth or health innovation setting were excluded. The mHealth developers' perspective was included by a scoping interview conducted with a representative of the medical technology company which initiated the research.

#### Professionals in Health Policy and Financing

In-depth face-to-face or telephone interviews (*n* = 6) were conducted with heath innovation managers at three different health insurance companies and policymakers at relevant government institutions, including the Directorate for Medicine and Medical Technology and the Program for Healthcare Innovation. Professionals were included based on their expertise to encourage or scale-up health innovations.

#### Primary Care Professionals (GPs, Practice Nurses, and Managers)

Eighty general practices (40 urban and 40 rural practices) were invited to participate based on random selection using “Zorgkaart Nederland” (map of Dutch general practices). Of these, nine expressed interest in participating. In total, 12 primary care professionals agreed to a short (25 min) semi-structured telephone interview (*n* = 12). Exclusion criteria for selection were: (1) practice nurses not providing care to chronically ill patients and (2) professionals having <6 months experience within the general practice. Participant characteristics are listed in Table 2.

**Table 2:** Characteristics primary care professionals' participated (*n* = 12).

		***n***
Sex	Male	3
	Female	9
Age	Median = 40	
	25–45	8
	45–65	4
Profession	GP	7
	Practice nurse	4
	Practice manager	1
Years of experience	<5	6
In general practice	5–15	2
	>15	4
Location (*n* = 9)	Urban (>2,000 inhabitants)	5
	Rural (<2,000 inhabitants)	4
Practice type (*n* = 9)	Solo	1
	Duo	2
	Group (>3 GPs)	6

#### Chronically Ill Patients

Participants of the focus group (*n* = 6) were recruited through the researcher's personal network by sending out an information leaflet. Those interested to participate were provided with additional information about the research and sampling procedure. People were eligible to participate if they suffered from a chronic disease and were Dutch speaking. For example, patients suffered from asthma, diabetes mellitus (type 1 and type 2), and arrhythmia. The participants' ages ranged from 17 to 83 (median 45) and had the diagnosis for 4–25 years.

### Data Collection

All participants were made aware of the nature and objectives of the research and gave written informed consent prior to the interview. Interviews lasted between 25 and 60 min. Probing techniques were used to explore perceptions and experiences. Interviews and the focus group were audio-taped and transcribed verbatim. Only the researcher had access to audio-recordings and transcripts which were treated with the strictest confidentiality and safely stored. Participants were registered anonymously by changing the name into a code.

At the start of the interview or focus group, the respondent(s) and interviewer reached consensus on what mHealth solution was referred to in response to interview questions. An example was provided by the interviewer of a smartwatch used by the patient to continuously self-monitor health data, such as heart rate monitoring and physical activity levels. The smartwatch can provide feedback to users on goal progress, send medication notifications and enable communication with Healthcare providers.

Exploratory interviews were conducted face-to-face. These interviews were held: (1) to identify the stakeholders engaged in future integration of mHealth, (2) to assess the level of support for mHealth integration, and (3) to map a stakeholder's position. Interviews were semi-structured and followed a topic guide based on the WHO stakeholder analysis guidelines (28). Participants reflected on issues as power, influence, motivation, resources, and interests in describing the role and position of key stakeholders. Additional documents, such as regulations, policies, and research reports mentioned as highly relevant by the participants were also included as data sources.

In-depth semi-structured interviews with professionals in health policy and financing were carried out face-to-face in the workplace (*n* = 2) or over the telephone (*n* = 4). Respondents were asked about the barriers and facilitators in the integration of mHealth in primary care. In addition, professionals were encouraged to think of strategies to ensure the scale-up of mHealth in primary care.

Primary care professionals participating in semi-structured telephone interviews were given the same core questions on exploring barriers and facilitators and formulating strategies to mHealth adoption. However, they focused on the barriers and facilitators they (expect to) encounter or experienced when implementing mHealth solutions in their practice. Additionally, primary care professionals were asked to provide socio-demographic information and to describe their workplace, such as practice type and patient population served.

A focus group was held to support participation and to establish a dialogue between patients to exchange experiences in managing their chronic illness. Initially, a heterogeneous focus group of patients and practice nurses was planned. However, it was not feasible for health professionals to join the focus group. In line with the literature, reasons provided were a lack of time, no (financial) incentive, not able to participate due to the many requests to join research studies (22, 29). Therefore, their perspectives obtained in the interviews were shared in the focus group and discussed among patients. During the focus group, the implications of a future integration of mHealth in primary care on patient self-management were discussed. In addition, participants jointly formulated the needs to make integration successful and identified barriers to the process of integration.

### Data Analysis

A number of methods were applied to improve the rigor and credibility of the research. First, the thematic analysis was conducted iteratively to reflect on transcripts and to verify (initial) findings in further interviews. Second, a member check of synthesized data took place by adding a validation step in which participants of exploratory interviews reflected on the results of the stakeholder analysis. Third, the researcher asked regularly for feedback after interviews and the focus group on the research process and whether participants were satisfied with (intermediary) outcomes. Moreover, triangulation was established by using various research methods—document analysis, interviews, and a focus group. Both written and oral data sources were used, and the study included a large and diverse sample of participants. Transcripts of audio-recordings were analyzed using the data analysis software program MAXQDA version 12.

#### Stakeholder Analysis

Identified stakeholders were grouped under six headings: government, research community, private business, civil society, health consumers/patients, and healthcare providers. As the stakeholder component was based on preliminary explorative research inductive codes were of prime importance to generate categories of information. Nonetheless, the stakeholder analysis was informed by all data gathered. During interviews, informants estimated the relative influence of each stakeholder on integration (power) and to what extent a stakeholder is affected by integration or held accountable in the process of integration (interest) (27, 30). In a second step, the power-interest grid was used to assess stakeholders' levels of influence and position toward mHealth integration at the time of the fieldwork (2017) (30).

**Figure 3:**
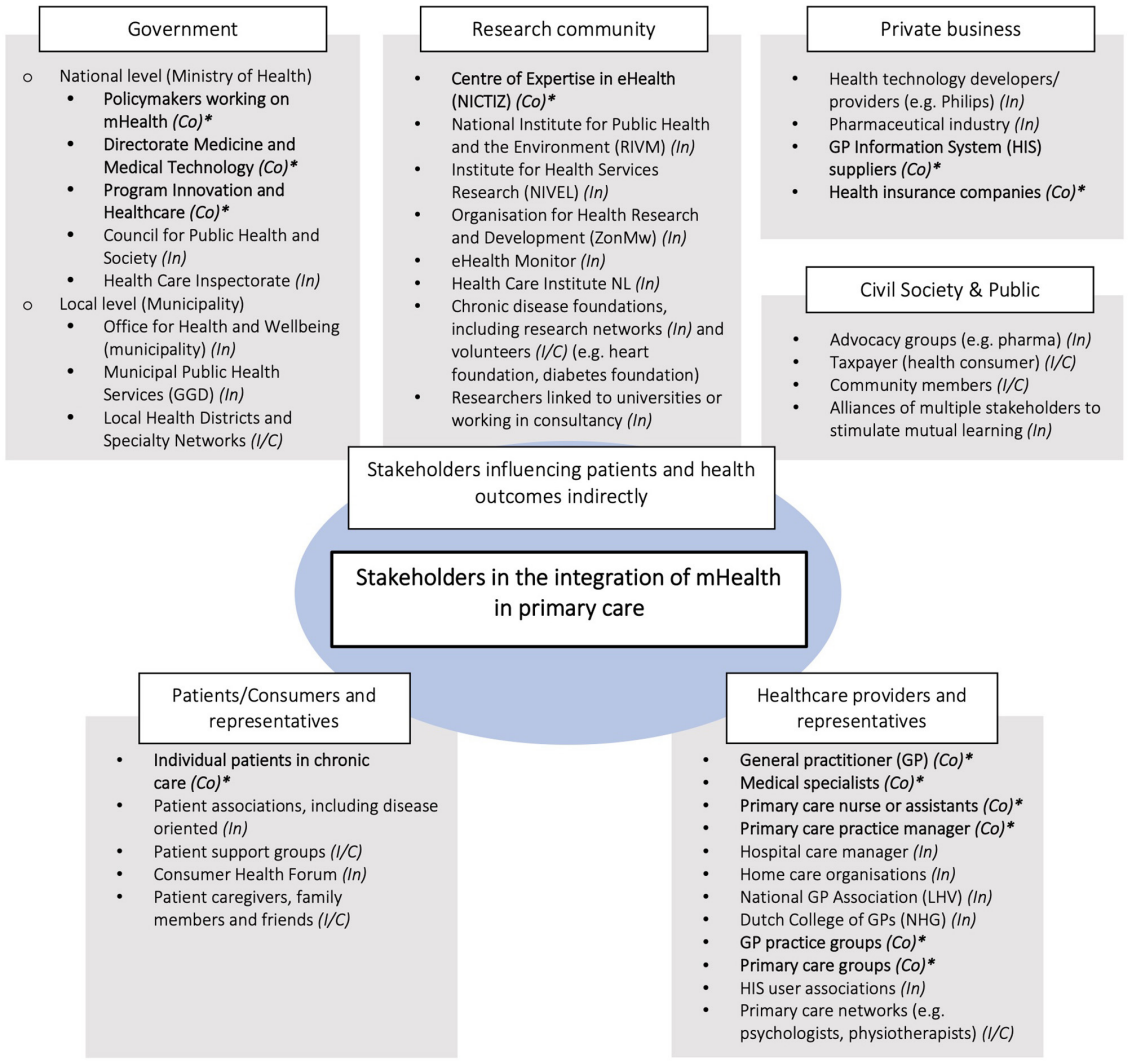
Stakeholder identification map. *Estimated power of stakeholder in terms of control *(Co)* (also, influence *(In)* and interest/concern *(I/C)*). Stakeholders regarded as having control *(Co)* over the adoption process are key stakeholders written in bold letters.

During the exploratory interviews, informants identified 42 stakeholders across the health system (Figure 3). They can be categorized in three main groups:

1. individual patients/consumers and their representative organizations.
2. healthcare professionals interacting with patients (e.g., GPs, practice nurses, medical specialists), and their professional organizations/associations.
3. institutions and organizations not directly in contact with patients/consumers, but able to affect their health (e.g., governmental institutions, health insurers, mHealth providers).

The first two groups comprise the end-users of mHealth solutions. They are directly affected by the potential adoption of new mHealth initiatives. The third group has the ability to influence the integration of mHealth in the health system. They considered their role mainly as a facilitator; providing knowledge and (financial) resources contributing to the functioning of the health system.

Of all 42 stakeholders identified, 13 were considered to have “control” over the adoption process, 22 to have “influence” and seven to have an interest or to be concerned about the situation (Figure 3). All 13 stakeholders in the “control group” were identified as key stakeholders based on their position to steer (or obstruct) successful integration. Box 1 illustrates the diversity in perspectives on who to involve in the integration process. Their characteristics are listed in Table 3.

Box 1 **Selected quotes regarding key stakeholders.**

**Quote 1:** “*It is important to include the Dutch expert organization on eHealth as they set the standards for health information exchange. These specifications are necessary for developers of mHealth to build high quality solutions.”* (Representative GP association)

**Quote 2:** “*You need to have the GP information system developers around the table. Some of these providers have quite a market share and financial assets. Changes to the system, such as allowing patients to access their own health data, needs to be discussed first and agreed to with the provider.”* (Representative patient association)

**Quote 3:** “*We [health insurer] believe the GP should be the ambassador of mHealth solutions for primary care. As the main provider of primary care, the GP is in a position to implement and offer mHealth solutions to patients.”* (Innovation manager health insurer 1)

**Table 3:** Key stakeholder characteristics around the integration of mHealth in primary care.

**Abbreviation**	**Stakeholder**	**Characteristics**				
		**Involvement in the!!break integration of mHealth**	**Interest**	**Influence/!!break power**	**Position**	**Impact integration on actor**
P	Individual patient in chronic care	Potential user of mHealth solutions	High	Low	Mostly supportive	High
MS	Medical specialist	Secondary care to chronically ill patients; due to a loss of income not likely to refer patients back to primary care	Low	Medium-high	Likely to be opposed	Low
GP	GP	Potential user and/or promotor of mHealth	Medium	Medium	Mixed	Medium
N	Practice nurse	Potential user of mHealth	High	Low	Mostly supportive	High
M	Practice manager	Providing support to GP on management tasks; potential promotor of mHealth	Medium	Low-medium	Likely to be supportive	Medium
GPGr	GP practice groups	Collaboration of GPs in group practices; potential users and/or promoters of mHealth	Medium	Medium	Mixed	Medium
PCG	Primary care group	Representing interests GPs; establishing contracts with insurers on behalf of GPs; potential promotor of mHealth	Medium	Medium-high	Likely to be supportive	High
PM	Policymaker (MoH)	Developing and shaping policies on mHealth	Medium-high	Low-medium	Supportive	Medium
PIH	Program Innovation & Healthcare	eHealth policy formulation and support; facilitator of stakeholder collaboration	High	Medium	Supportive	Medium
DMT	Directorate Medical Technology	Assessing and approving health technologies on accessibility, quality and safety	Medium	Medium	Supportive	Medium
NIC	Centre of Expertise in eHealth (NICTIZ)	Policy support, particularly by setting health data exchange standards	Medium	Medium	Likely to be supportive	High
HIS	GP information system developer	Development and control of information systems in primary care; monopoly market position	Low-medium	High	Likely to be opposed	High
I	Health insurer	Pays for health care; searching for solutions to keep health care affordable	High	High	Supportive	Medium-high

#### Health System Analysis

To identify and analyse barriers and facilitators in the integration of mHealth a directed content analysis, including deductive and inductive coding was used. Coding was based on a modified version of Murray and Frenk's framework for assessing the performance of health systems (19, 20). First, the deductive coding method allowed to see where in the four functions of the health system barriers and facilitators exist [Figure 2; (19)]. Subsequently, an inductive approach was used to explore relevant concepts that did not fit the framework and needed to be assessed in broader perspective. This contributed to an in-depth understanding of barriers and facilitators.

## Results

This section presents the identified stakeholders and provides an analysis of the barriers and facilitators in the integration of mHealth. In discussing mHealth solutions participants referred to examples they learned of but were not necessarily working with professionally or using in daily life. Examples participants considered relevant are mobile phone applications to promote healthy behavior, a smartwatch for self-monitoring, and medication reminders through SMS. In contrast, eHealth applications were frequently used among the end-user groups, such as an online tool to plan a GP appointment or request (repeat) prescriptions.

### Stakeholder Analysis

**Figure 4:**
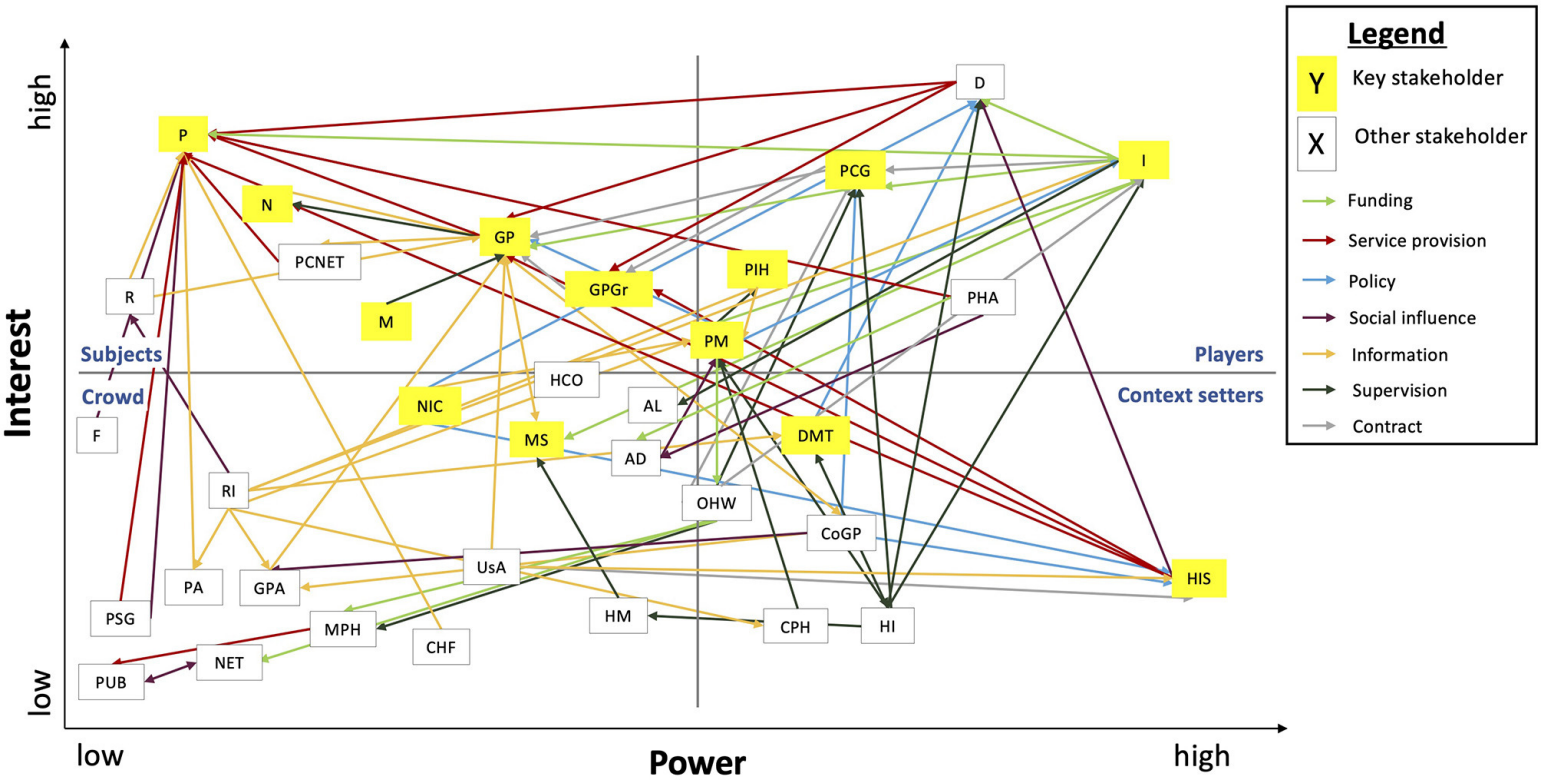
Power-interest grid of stakeholders engaged in the integration of mHealth. AD, advocacy groups; AL, alliances; CHF, consumer health forum; CoGP, college of GPs; CPH, council of public health; D, mHealth developer/provider; DMT, directorate medicine and medical technology; F, family and caregivers; GP, general practitioner; GPA, national GP association; GPGr, GP practice groups; HCO, home care organization; HM, healthcare manager; HI, healthcare inspectorate; HIS, GP information system suppliers; I, health insurer; M, primary care manager; MPH, municipal health services; MS, medical specialist; N, primary care nurse; NET, local networks; NIC, expert organization eHealth (NICTIZ); OHW, office health and well-being (municipality); P, patient; PA, patient association; PCG, primary care group; PCNET, primary care networks; PHA, pharmaceutical industry; PIH, program innovation & health care (MoH); PM, policymaker; PSG, patient support group; PUB, public; R, researcher; RI, research institute; USA, GP information system user association.

The analysis of exploratory interviews indicated that most stakeholders were supportive of the integration of mHealth in primary care, although differences exist in their level of influence (Table 3). The power-interest grid was used to compare the positions of individual stakeholders and the relations among them (Figure 4).

#### Supportive Stakeholders With High and Medium-High Influence

As illustrated in Table 3, key supportive stakeholders with high and medium-high influence are primary care groups and health insurers. Primary care groups are legal entities owned by GPs in a particular region (31). They differ in size from 4 to 150 GPs. Approximately 80% of Dutch GPs was part of a care group in 2014 (32). Core functions of care groups are to coordinate chronic illness care and to negotiate a fixed fee per patient with a health insurer. To receive such a bundled payment requires a contract between a care group and health insurer (32). These contracts may include budget reservations for health innovation. One interviewee illustrated the process of negotiation.

“*It is open to discussion to use health technology, [say it is] A, B or C. This is determined in the contract between the health insurer and healthcare provider. Health insurers usually push the discussion toward health technologies they find useful to purchase.”* (Policymaker Directorate Medicine and Medical Technology)

Care groups were identified as potential promotors of mHealth integration. They are interested in implementing health technologies, such as mHealth, for three reasons: (i) to reduce the workload of health professionals; (ii) to increase the quality of care; and (iii) to meet the expectations of the patient population they serve. However, care groups, but also individual GPs might experience difficulties in receiving funding for mHealth solutions. Ideas for innovation are assessed based on criteria of improved quality of care, reduced healthcare costs, and increased satisfaction among patients. Unless health technologies fulfill these criteria, health insurers are hesitant to provide funding for implementation.

“*Only when the effectiveness of a mHealth solution is demonstrated by evidence we might step in. We won't provide funding if we think it is a risky investment.”* (Innovation manager health insurer 1)

Although their influence is substantial, health insurers describe their role as “facilitating”. This facilitating role is portrayed two-fold. First, health insurers encourage primary care providers to think about innovative ways to deliver affordable, high-quality care within existing budgets. In addition, health insurers may help realizing new ideas by revising the contract or referring to external sources of funding, such as funds at regional governments.

“*We ask GPs to share their thoughts on how to enhance patient-centered care at an affordable price. Once there is a good idea, we discuss what changes to the contract are necessary for GPs to realize their plans.”* (Innovation manager health insurer 2)

Second, a facilitating role is observed toward the clients of health insurers—the insured health consumer. A health insurer's main interest is to create best value for money and keep premiums low. mHealth has the potential to provide health services at decreased costs (33, 34). Particularly with regard to disease prevention, health insurers will actively promote the supply of effective mHealth solutions. This is either done by providing guidance to clients on how to purchase available mHealth services, or through collaboration with mHealth enablers. This collaboration may lead to health applications specifically designed for clients to prevent or manage chronic diseases.

#### Supportive Stakeholders With Medium/Medium-Low Influence

Supportive stakeholders with medium influence were mainly working in government agencies under the Ministry of Health, including the Program of Innovation and Healthcare, the Directorate Medicine and Medical Technology and the Centre of Expertise in eHealth (NICTIZ). Departments of the Ministry of Health prepare and implement policies and programs to support the uptake of innovation in Health care. Policy support by providing research evidence on eHealth and health innovation is a key role of NICTIZ. Here, the term eHealth refers to the use of Internet and related information technologies to communicate health-related information and deliver interventions. They are an important stakeholder in the integration of mHealth in primary care as they set the standards for health information data exchange and are responsible for the eHealth application already used in primary care, such as online tools for making a GP appointment (Box 1, quote 1).

Whether more focused on conducting research or formulating policy, these governmental stakeholders have in common that they want to facilitate knowledge exchange between various stakeholder groups. A policymaker at the Ministry of Health illustrates this role.

“*We [Ministry of Health] do not envision a top-down approach in integrating mHealth in chronic care. Rather we bring stakeholders together, including representatives from health insurers, patient and physician organizations, industry and research to discuss ideas for implementation.”* (Policymaker Ministry of Health).

#### Supportive Stakeholders With Low Influence

Supportive stakeholders who agree that mHealth should have a profound role in chronic care delivery but have less influence on the integration of mHealth in primary care, include chronically ill patients and practice nurses. They are the main potential users of mHealth technologies. Among all end-users, chronically ill patients expressed the highest interest in mHealth adoption. This group noted some perceived benefits of mHealth, such as quick and easy communication with healthcare providers, and increased patient autonomy.

“*mHealth can make daily life easier, because it enables quick communication with my doctor in a convenient way. I can have an answer to my questions within minutes.”* (Patient 1, age 49)

“*Having an overview of my blood sugar level on my phone would allow me to have better insight in my health. It can help to understand blood sugar fluctuations, so I can focus on improvements.”* (Patient 2, age 17)

Despite perceived benefits, representatives of patient organizations emphasize the diversity in perspectives with regard to mHealth among patients. Particularly adolescents and young adults support the use of mHealth solutions in primary care, while the older patient population is more skeptic. This contrast was explained by the extent to which patients are familiar with mHealth technologies and able to use them in managing their health. Nonetheless, patients encourage the availability of relevant and easy-to-use mHealth solutions.

Another important end-user in this respect is the practice nurse. As the main provider of chronic care, they have a high interest in the adoption of mHealth. Practice nurses are responsible for the regular check-ups of chronic care patients and supervise in chronic disease management. Faced with a substantial (administrative) workload, practice nurses perceive mHealth as a supportive tool which can potentially save time.

“*It [mHealth] saves time. My patients will be able to contact me directly, instead of talking to a practice assistant first.”* (Practice nurse, urban group practice).

### Barriers and Facilitators

Findings on the barriers and facilitators in mHealth integration are structured along the four health system functions: stewardship, financing, service provision and resource generation. Additionally, the thematic analysis revealed several themes that do not fit one of the four functions and are therefore analyzed in wider perspective of the health system.

#### Stewardship

Stewardship aims to set, implement and monitor the rules for all actors (healthcare providers, payers, and consumers) within the health system. In this domain, respondents indicated barriers related to an inadequate set of core standards, and difficulties in informing end-users on mHealth. Using reflexive learning approaches including multiple stakeholders and gathering evidence were seen as facilitating factors.

##### An inadequate set of core standards

Respondents identified a lack of specific regulation on mHealth to ensure health information obtained by mHealth applications complies to privacy and data security standards. End-users suggested to define a set of clear principles applied to all use of patient data and to all data controllers to guarantee the protection of mHealth data. The need for universal standards was also observed in enabling health information exchange.

“*Making sure mHealth data is not used for commercial purposes is something we [the government] can actively promote by setting the rules.”* (Policymaker Directorate Medicine and Medical Technology)

Currently, patients are not able to view, download, and transmit their own health information from an electronic health record (EHR) to a personally controlled health record. However, recently the law “electronically processing of patient data” was implemented, stating that patients should have access to their own health information by 2020 (35). Allowing patients access to their own health information is a first step enabling mHealth implementation. However, despite new regulation, barriers exist in the interoperable exchange of health data. Healthcare providers cannot directly transfer patient data received from sensors or applications to an EHR. This prevents (self-measured) mHealth data to be saved in health records. There is a need to produce a set of core standards and specifications that enable EHRs to communicate seamlessly.

##### Difficulties to inform on mHealth

In addition to barriers related to the legal framework, respondents identified obstacles in informing end-users. A key role of the Ministry of Health is to inform and make health consumers and professionals aware of the possibilities of mHealth. The validation of mHealth applications is necessary to prevent false claims made by mHealth developers on the effectiveness of certain mHealth products or services. However, established research methods aiming to measure effectiveness lag behind.

“*It is not easy to validate and measure the effectiveness of over 200 000 health applications. Traditional methods of clinical trials are not appropriate anymore. Clinical trials usually take long. By the time evidence is produced the technology is outdated.”* (Policymaker Program on Healthcare Innovation)

Collaboration with mHealth developers is necessary to communicate standards on the quality criteria to be followed during app development. Moreover, appropriate assessment methodologies are needed to measure validity and reliability of mHealth apps in order to provide recommendations to consumers.

##### Encourage reflexive learning

A recurrent theme within the dimension stewardship was the lack of collaboration between stakeholders. Respondents identified the enhancement of interaction between stakeholders as a key facilitator in mHealth adoption at greater scale. More specifically, the Ministry of Health was appointed to have an important role in this process of bringing stakeholders together.

In steering collaboration, it was suggested to encourage stakeholders to learn about each other's perspectives and experiences on how mHealth can be of added value in chronic care delivery. Reflexive learning approaches may support this vision of realizing collaboration in mHealth adoption. A policymaker at the Program for Health Innovation reflected on this.

“*Not all parties are willing to share their insights to come to collaboration. They mainly think from their own perspective. The scale-up of mHealth can only be achieved when stakeholders step out of their comfort zone and develop a shared vision to integrate solutions”* (Policymaker Program on Healthcare Innovation).

##### Gathering evidence

There is a need for scientific evidence on the effectiveness of mHealth solutions. Evidence-based information would allow stakeholders to make informed decisions in considering the use or reimbursement of mHealth solutions. A possible explanation for the lack of evidence is the large, non-regulated (international) market for digital health technologies, making it hard to assess what technologies can be successfully adopted. Due to a lack of time and resources, GPs are hesitant to adopt mHealth services without proven effectiveness.

“*We [GPs] won't immediately implement the newest technologies. The technology should prove itself and earn our trust.”* (GP, rural group practice)

A GP described how evidence may be gathered.

“*Ideally scientific research shows the effectiveness of a mHealth solution. If this is not possible, respected physicians need to testify on its added value.”* (GP, urban duo practice).

#### Financing

Health system financing comprises a range of processes, including revenue collection, fund pooling, and purchasing. Within this dimension, specific focus lies on payment structures in primary care. Currently, there are no specific funds available for mHealth adoption by GPs in the Netherlands.

However, based on the guidelines for financing eHealth solutions, GPs have several opportunities to mobilize funds within the existing payment system [Box 2; (36)]. Within these payment structures, the main barriers were a lack of time and resources to ensure financing for mHealth, and difficulties in applying for (extra) funding. Presenting a good revenue model for the implementation of mHealth solutions was perceived as a facilitator.

Box 2 **Guidelines for financing eHealth solutions (33).**

Mobilization of funds for health innovation within existing primary care payment system:

- the capitation fee can be used to build an online health environment to facilitate communication between patients and primary care professionals;
- remote consults can be reimbursed as regular, face-to-face consults;
- revenues collected to finance chronic care can be spend on solutions for the remote delivery of chronic care or self-management programs;
- health insurers and Healthcare providers may have additional negotiations about reimbursing or rewarding the use of digital health solutions in delivering primary care services.

##### A lack of time and resources to establish financial flows for mHealth

GPs mentioned that existing budgets were not satisfying to realize mHealth service provision. They felt compelled to apply for additional funding which was identified as a time-consuming and resource intensive process, particularly for stand-alone practices. While in group practices a manager takes on administrative and financial tasks, solo-practicing GPs must constantly monitor their financial resources.

##### Difficulties in applying for funding

GPs indicated the health insurer as the first contact point to turn to for financial support to implement mHealth. Applying for funds was reported by the majority of GPs as a time-consuming and inconvenient process. Another barrier to the application process was the request of health insurers to have access to patient data. GPs felt disturbed to allow access to patient data and were hesitant to do so.

“*I tried to apply once. [.] However, I withdrew my application as I was pretty annoyed by the unwieldly and bureaucratic system, leaving me with a high administrative burden.”* (GP, rural solo practice).

##### Thinking about a revenue model

Health insurers noted a better chance of receiving funding, if a GP presented a long-term revenue model. To be able to do so, it is important to remove barriers from the application process, making it easier and less time-consuming to apply for funding. Moreover, GPs indicated a lack of knowledge to develop a smart financing model. Better cooperation between GPs and health insurers is needed to make existing budgets fit for implementing new innovations.

#### Service Provision

By service provision is meant personal health services directly consumed by the health system user which can be preventive or disease management services in which mHealth might assist. Despite perceived benefits, negative perceptions of end-users toward mHealth may obstruct adoption. Concerns were expressed on a changing physician-patient relationship, and risk of misinterpreting health data. Facilitators were associated with providing high quality and relevant mHealth solutions.

##### Risk of losing personal contact

The risk of losing physical contact was perceived as a barrier to using mHealth solutions. GPs indicated that face-to-face consults help to pick up signs relevant to the course of treatment, but without this interaction remain under the surface. Moreover, particularly older patients preferred personal contact with their GP.

“*I would rather go to the GP than communicate remotely. I like to be there and to have a chat.”* (Patient 3, age 83)

“*Why would you want to communicate remotely, if you can talk with each other directly by paying a visit? An inconvenient way to communicate with your doctor, I believe.”* (Patient 4, age 58)

However, this view was not shared among all end-users. Using mHealth services was also associated with perceived benefits, such as a reduced workload and saving time. A GP using mHealth services commented.

“*I prefer online interaction as it is quicker. I settle a lot online, which means I have less patients who visit me in person. While a GP consult normally is 10 minutes, in my practice I can spend 20 minutes on a face-to-face consult. It is more relaxed and satisfying than it used to.”* (GP, urban group practice)

Generally, a common notion exists that mHealth is an additional tool which is not able to replace physical contact. When necessary or preferred a face-to-face consult should be possible.

##### Risk of misinterpreting health data

Another barrier identified by primary care professionals was the concern to misinterpret mHealth data. The risk of misinterpretations was mainly observed in assessing self-measured data. GPs and nurses said to have difficulties to make sense of the data patients collect. More specifically, the quality of self-measured data was sometimes doubted as patients need to carry out self-measurements at the right time and under the right circumstances, following procedures correctly to ensure data validity.

As a result of poor data quality, healthcare providers might be inclined to see a patient in person to conduct the test themselves. Therefore, it was suggested to devote time of practice nurses on informing and educating patients on the use of mHealth services as it has the potential to save time in future consults.

##### Good working mHealth solutions

A facilitator in the service provision domain was to create high-quality mHealth solutions tailored to the needs of end-users. It was suggested that mHealth developers focus on the usability and user-experience of end-users. By usability was meant applications that are easy to use and potentially timesaving, while user-experience included the alignment with daily life situations and having an added value to the care process. Subsequently, it was suggested among end-users to harmonize the use of validated apps. This implies the possibility to align high-quality applications in one portal, that can be easily accessed by end-users.

#### Resource Generation

Health systems include a diverse range of inputs to provide health services, such as human resources, equipment and knowledge. Barriers within the function resource generation were associated with the knowledge, skills and attitudes of stakeholders. Education was mentioned as a facilitator to overcome barriers.

##### A lack of knowledge

GPs mentioned they are poorly informed about the possibilities of mHealth. Moreover, if they were informed, GPs stated they have insufficient knowledge on how to adopt and use health technologies in their practice. A graduate of the GP specialty training program explained.

“*My study taught very little about entrepreneurship. Only a few GPs like the entrepreneurial aspect of their job, but most of them don't. They want to focus on providing health services.”* (Graduate in Medicine, GP specialization)

Knowledge on the possibilities of mHealth, but also operational support in how to implement mHealth services is necessary to realize adoption.

##### A lack of digital skills

Another obstacle for end-users is a lack of digital skills to work with mHealth solutions. Particularly, older generations may experience difficulties in using mHealth technologies as they need to switch to a new way of working. A lack of digital skills results in extra time needed to enter or process data. It was suggested to develop mHealth technologies which are user-friendly and very easy to use to keep the need for digital skills at a minimum.

##### Negative attitudes of stakeholders

Attitudes of stakeholders refer to constructs defined by values stakeholders hold. The attitude of GP information system developers was identified as a barrier to the adoption of mHealth.

“*GP information system controllers hold the key to providing access to their system by other providers. If access is not granted to certain mHealth technologies, we cannot easily transfer the collected mHealth data to our GP information system.”* (Practice nurse, urban group practice)

The lack of motivation among GP information system developers to optimize the systems they produce obstructs quick information exchange between the GP information system and the mHealth technology. Consequently, healthcare professionals spent extra time entering (self-measured) patient data in the EHR. Therefore, partnerships were suggested between mHealth technology developers and GP information system owners to ensure alignment of services. The government has a crucial role to stimulate this collaboration or might even intervene in the market to encourage mHealth adoption.

The attitude of GPs was also frequently discussed among respondents as a barrier in the integration of mHealth in primary care. First, GPs are afraid of resistance toward the implementation of new technologies by their patient population. Furthermore, GPs doubt their patient population to be capable of working with technological solutions. Particularly, GPs working in rural areas or serving a patient population with a low social economic status (SES) mention this problem. Second, GPs are hesitant in taking initiative. They find it hard to embrace and accept a new way of working. The little drive for entrepreneurship in combination with a lack of time and resources led to an attitude of “wait and see” instead of taking initiative to innovate.

##### Education

Proving education on how to use and implement technologies was mentioned as a facilitator in tackling barriers related to knowledge, skills and attitudes. Currently, curricula devote little time to the entrepreneurial aspects of being a GP. Reserving time in the curriculum of prospective primary care professionals to teach on topics as management and health technology adoption will increase their knowledge of the possibilities to innovate in general practices.

#### Overarching Health System Factors

The analysis revealed themes that did not fit any of the four health system functions. Instead, they were analyzed at a higher, overarching level. One barrier identified in this area related to system design: a production-stimulating system leading to a wrong financial impulse among physicians. A facilitating factor enhancing the adoption at health system level was the inclusion of stakeholders and relates to the responsiveness of the health system.

##### A production-stimulation system

The production-stimulating impulse in the health system causes healthcare professionals to provide more service. This is encouraged by a fee-for-service payment system. While detaining chronic care patients in primary care is saving healthcare costs, medical specialists are not likely to refer patients back.

“*The financial impulse is wrong. Medical specialists have no reason to send chronic care patients back to primary care, because they earn money for treating quite healthy people. It is cheaper and better for the continuity of care to help these patients in primary care.”* (Innovation manager health insurer 2)

Medical specialists find it hard to accept a loss of income when referring (chronically ill) patients back to primary care. However, money can be better spent in primary care. Therefore, gradually reducing the budget for medical specialists who refer patients back and providing more budgets to GPs taking care of these patients is necessary to make this transition.

##### Inclusion of stakeholders

At health system level, a facilitator was to ensure stakeholder inclusion in mHealth integration. Inclusion was observed in two areas. First, including end-user perspectives from the start of product development will ensure mHealth services meet the needs of end-users. This co-design of mHealth technologies contributes to high-quality and relevant solutions that are more likely to be used and adopted by end-users. Second, respondents suggested to create a business case. This implies collaborations of multiple stakeholders engaged in mHealth integration to establish a thorough adoption strategy. A beneficial business case represents common interests, by including advantages for all stakeholders, such as increased affordability of care, high quality of care, and consumer satisfaction.

## Discussion

This research extends our understanding of how to integrate mHealth solutions into primary care. On the one hand, end-users of mHealth, i.e., primary care practitioners and patients, seem to have a supporting or mixed attitude toward integration of mHealth. On the other hand, several powerful stakeholders, including primary care information system developers and medical specialists are likely to show resistance or a lack of initiative toward mHealth integration. Key barriers to integration perceived by stakeholders included: a lack of interoperability with existing information systems, difficulties in obtaining funding for implementation, and limited readiness of general practices to change. In contrast, key strategies to facilitate integration were collaboration between stakeholders, and incentives for pioneers. The findings indicate that mHealth integration is challenged at different levels, including higher health system level barriers, organizational level barriers and technical features as barriers.

At system level, the lack of universal standards obstructs the interoperability between mHealth devices and existing primary care information systems. The role of standardization has been a long-recognized topic in eHealth and mHealth (37, 38) and has been reported earlier in policy documents (39, 40). However, our findings indicate that this is still a significant barrier in integration of mHealth. Previous studies have shown that addressing interoperability by establishing a regulatory framework can be favorable to the success of mHealth implementation (15, 41, 42). Regulation is needed to create an infrastructure to facilitate information exchange and ensure all information systems adhere to interoperability standards. This requires regular involvement and communications between the Ministry of Health, the Centre of Expertise in eHealth (NICTIZ), GP information system developers, and mHealth developers to set data standards and specifications. NICTIZ has a key role to ensure systems fulfill requirements prior to entering the market.

Another barrier that needs to be addressed at system level is the perceived difficulty in establishing budgets and ensuring financial flows for mHealth implementation. GPs indicated a lack of time and resources to transform existing budgets to fit mHealth costs. Meanwhile, insurers argued that uncertainty about return on investment led them to withdraw from providing funding for mHealth. This tense relationship has been reported by other studies and can be explained by the different interests and values these stakeholders hold (43–45). Whereas, GPs are concerned with providing acceptable, high-quality health services on a long-term basis, insurers' main concern is affordable Health care with return on investment in the short run.

Demonstrating evidence on return on investment is critical for insurers to reimburse mHealth services (46, 47). There is a need to define robust metrics for measuring the efficacy and (cost-) effectiveness of mHealth services (40, 46). Rather than conducting traditional clinical trials, such studies could use practical evaluation methodologies including clinical, patient-reported and economic outcomes (46, 48–50). An example is the use of a micro-randomized trial design to assess the causal moderated effect of intervention components by using standardized effect sizes (48). This design allows comparison of the effectiveness of different intervention components. A proposed solution would be to combine evidence on effective intervention components and a pragmatic approach when designing or adapting mHealth solutions to allow conditional reimbursement approval (20, 46, 47, 49). This financing model serves to encourage practice-based interventions, while decreasing the risks for health insurers.

At organizational level, barriers were found in the practice “readiness” for implementation. Overall, primary care professionals were positive about mHealth adoption. However, some GPs indicated a lack of “sense of urgency” and motivation to implement mHealth solutions. One explanation is that GPs feel comfortable the way they operate their practice and are satisfied with the IT solutions they use. Pilot studies implementing mHealth technologies in primary care processes in Denmark and Sweden found healthcare clinics with a pre-existing culture of desire to provide care in a more modern way and attitudinal ethos of quality improvement to be more receptive to the introduction of mHealth services (50, 51). These characteristics, together with strong internal and external stakeholder collaboration, are essential to create an “implementation climate” for successful adoption (50, 51). Another explanation is uncertainty about whether mHealth solutions can meet expectations of saving time and maintaining high-quality care, and thus have an added value in delivering health services. Building end-user trust in mHealth solutions, preferably by providing evidence-based information on app credibility, is an important enabler to increase promotion of mHealth solutions by GPs (45, 52, 53). This includes information on perceived usefulness, ease of use, risks associated with accessing and communicating personal health data and a measure of trust in the developers of the mHealth technologies (54, 55).

With respect to technical features, end-users, including GPs, practice nurses, and chronically ill patients experienced a lack of time and technical skills to adopt mHealth solutions. To promote use and acceptance, respondents indicated that mHealth solutions need to be very easy to use, reflect meaningful functionality, and align with the context of an end-user's daily life and workflow. In line with findings from the literature, mHealth technologies perceived to be easily embedded in existing structures and timesaving have a better chance of being adopted (15, 44, 45, 54). GPs and practice nurses emphasized their limited amount of time; therefore, adding extra mHealth-related tasks to their workload would be undesirable.

By exploring health system constraints and opportunities, and stakeholder views two main strategies emerged to steer mHealth integration. A first strategy is stimulating co-design of mHealth technologies. It has been demonstrated that end-user involvement in the development of mHealth solutions is crucial to support acceptance and adoption of new technologies (42, 47, 56). This requires a thorough examination of end-user needs and capabilities to use ICT equipment. Continuous feedback loops in the development process help to assess the level of support for adoption and lead to tailored solutions (41, 51).

A second strategy is for the government or health insurer to provide incentives for pioneers to make mHealth adoption more attractive. Several studies found strong stakeholder collaboration in which financial support is assured to initiate and energize the mHealth adoption process (47, 52, 57). A crucial facilitator is therefore to create collaborative platforms including key stakeholders, such as end-users, health insurers and industry players to make a sound business case shaping the adoption process. The ILA methodology used in this study is a good example of an iterative approach to involve and analyze stakeholders in the integration process. The learning cycles presented in this study form the basis for follow-up research to continue studying and supporting the integration process.

Regional care groups may act as a pioneer as they have, compared to small practices, the organizational means for implementation. These care groups can use (conditional) funding to slowly introduce mHealth solutions in local general practices. An option would be to appoint a key person in the organization (e.g., the practice nurse specialized in chronic care) who familiarizes with the mobile technology at hand and is trained to use it. Previous studies show that healthcare professionals are more likely to accept adaptations in their workflow from someone they trust and is seen as important to the job (51, 55). This person can introduce the wider team to the app and gradually inform and educate patients in using mHealth services. By weekly evaluating the adoption process in existing team meetings, barriers can be quickly picked up and improvements made to optimize the process (20, 51). In this way, general practices implementing mHealth solutions should be viewed as “learning cases” and can stimulate others for change.

One of the strengths of this study lies in the use of a transdisciplinary approach. This facilitated the inclusion of different types of knowledge from a variety of stakeholders. Considering health system functioning and stakeholder views allowed for a comprehensive understanding of the mechanisms involved in mHealth integration. Moreover, the stakeholder analysis complemented this study by providing information on stakeholder interests and power which contributed to the development of a strategic view on future mHealth integration (28, 58). Through member checking by discussing results in post-interviews, a validation step was added. However, this was only done among participants of exploratory interviews. Another strength of this study is a whole-of-system approach to capture important cross-system relationships instead of solely focusing on several aspects of adoption.

This research has several limitations. A major limitation was the limited time available to establish learning cycles. During this research, only one learning cycle was completed by discussing the results of the stakeholder analysis in follow-up interviews. A second learning cycle, discussing mHealth integration among different stakeholder groups proved difficult. Another limitation is that the software developers and suppliers of mHealth solutions were not or weakly represented. Furthermore, due to a lack of time, GPs and practice nurses could not be included in the focus group. Previous research shows that lack of time and interest is a main reason for physicians not to participate in participatory research. Yet, their participation is considered crucial for implementation (22, 29). To minimize bias, their views were shared in the focus group to encourage reflection and learning. Another limitation was the small size of the focus group in which chronically ill patients from mainly rural areas participated. More extensive research is needed to have a representative and detailed view of patient perspectives.

It is recommended to examine how proposed strategies can be applied in practice. The cross-sectional nature of this study does not allow to see patterns of adoption change over time. Hence, in future studies, it would be interesting to gather longitudinal data to establish causal relations on what impact various determinants have on adoption over time. In addition, extending this research to other European countries representing a variety in health systems would complement the research as this study only focused on the Dutch context. Finally, further research is recommended to examine the effectiveness of the ILA methodology in facilitating mHealth adoption in health systems.

## Conclusion

This study shows that interests and values of stakeholders may contradict each other and have substantial influence on the potential integration of mHealth in primary care. Nevertheless, most stakeholders support the adoption of mHealth in primary care. Addressing barriers with regard to the legal, financial, socio-cultural and technical aspects associated with mHealth adoption is needed to steer integration. This complex array of factors obstructing the scale-up of mHealth calls for future integration strategies that encourage collaboration between multiple stakeholders. Although this study focuses on the Dutch case, and is therefore not generalizable, findings are transferable to contexts similar to the Netherlands, including features of strong primary care with GPs as gatekeepers and an advanced technological environment in Health care.

## Data Availability Statement

The datasets generated for this study are available on request to the corresponding author.

## Ethics Statement

The studies involving human participants were reviewed and approved by the Internal Committee Biomedical Experiments of Philips Research (approval no: ICBE-2-18916). The patients/participants provided their written informed consent to participate in this study.

## Author Contributions

EB conducted the research, including design of the research, data collection, data analysis, interpretation, and writing the article. TC supervised EB throughout the research process and provided essential feedback to the research process, critically revised the article, and gave the final approval of the version to be published.

### Conflict of Interest

At the time of study, EB was employed as a research intern at Philips Electronics Nederland B.V. as part of her training in Research Master in Global Health, Vrije Universiteit Amsterdam. TC served as a part-time consultant to Philips Electronics Nederland B.V. in 2016–2017. Apart from the internship role of EB and consultant role of TC, the authors declare no potential conflicts of interest with respect to the research, authorship, and/or publication of this article.
